# Polaron pair mediated triplet generation in polymer/fullerene blends

**DOI:** 10.1038/ncomms7501

**Published:** 2015-03-04

**Authors:** Stoichko D. Dimitrov, Scot Wheeler, Dorota Niedzialek, Bob C. Schroeder, Hendrik Utzat, Jarvist M. Frost, Jizhong Yao, Alexander Gillett, Pabitra S. Tuladhar, Iain McCulloch, Jenny Nelson, James R. Durrant

**Affiliations:** 1 Department of Chemistry, Centre for Plastic Electronics, Imperial College London, Exhibition Road, London SW7 2AZ, UK; 2 Department of Physics, Centre for Plastic Electronics, Imperial College London, Exhibition Road, London SW7 2AZ, UK; 3 SPERC, Physical Sciences and Engineering Division, King Abdullah University of Science and Technology (KAUST), Thuwal 23955−6900, Saudi Arabia

## Abstract

Electron spin is a key consideration for the function of organic semiconductors in light-emitting diodes and solar cells, as well as spintronic applications relying on organic magnetoresistance. A mechanism for triplet excited state generation in such systems is by recombination of electron-hole pairs. However, the exact charge recombination mechanism, whether geminate or nongeminate and whether it involves spin-state mixing is not well understood. In this work, the dynamics of free charge separation competing with recombination to polymer triplet states is studied in two closely related polymer-fullerene blends with differing polymer fluorination and photovoltaic performance. Using time-resolved laser spectroscopic techniques and quantum chemical calculations, we show that lower charge separation in the fluorinated system is associated with the formation of bound electron-hole pairs, which undergo spin-state mixing on the nanosecond timescale and subsequent geminate recombination to triplet excitons. We find that these bound electron-hole pairs can be dissociated by electric fields.

## Abstract (web summary)

The spin dynamics at organic donor–acceptor junctions is critical in determining charge generation and recombination in devices, but the detail is still unclear. Here, Dimitrov *et al*. observe singlet–triplet spin mixing at nanosecond timescales, which competes directly with free charge separation.

## Graphical abstract

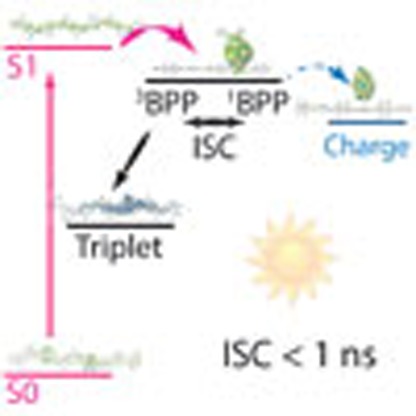

Electronic excited states in organic semiconductors used for optoelectronics and spintronic applications are Coulomb bound electron-hole pairs, called excitons with a total spin of zero or one. The triplet excitons (spin one) are typically non-emissive, less energetic and have longer lifetimes than the singlet excitons. They are generated via intersystem crossing from photogenerated singlet excitons, which is a vibrationally mediated process involving a spin flip enabled by spin-orbital interactions. In addition, charge pair recombination can also lead to the generation of triplet excitons. For example, luminescence from organic light-emitting diodes (LEDs) is generated by the recombination of electrically injected charges that yield emissive singlet excitons, with the parallel recombination pathway to non-emissive triplet excitons being a key limitation to device efficiency^1^,^2^. In organic solar cells (OSC), the direct intersystem crossing from photogenerated singlet excitons is typically suppressed by efficient and ultrafast singlet exciton dissociation at the donor/acceptor (D/A) heterojunction. Charge recombination can, however, lead to the formation of triplet excitons^3^,^4^,^5^,^6^,^7^,^8^,^9^,^10^,^11^,^12^,^13^,^14^,^15^,^16^,^17^, most likely via back-electron transfer from electron-hole pairs with a triplet multiplicity, in which the electron resides on the acceptor and the hole on the donor^3^,^4^,^18^.

The behaviour of coulombically attracted electron-hole pairs formed across D/A junctions is the subject of current debate, including in particular their role in mediating charge separation and recombination in OSC. They may be formed either as a result of exciton separation or charge pair association^19^,^20^,^21^,^22^,^23^,^24^. During the latter process, already separated charges with uncorrelated spins will populate both singlet and triplet electron-hole pairs in a 1:3 ratio. Back-electron transfer from such triplet electron-hole pair states can lead to a population of energetically lower lying (than the electron-hole pair state) triplet excitons^3^. The timescale for such a triplet exciton population is expected to be limited by the rate of nongeminate charge recombination, often on the range of submicrosecond to milliseconds^3^. Coulombically bound electron-hole pairs typically exhibit relatively short (<10 ns) lifetimes. As such it is not clear whether singlet to triplet conversion can occur within these states. Spin-state mixing of such electron-hole pairs generated by exciton separation at polymer/fullerene interfaces has previously been invoked to explain the observation of triplet exciton generation in such blends^4^,^6^,^7^,^13^,^14^. However, a recent study has suggested that the inability of such bound states to undergo singlet-triplet mixing may be a key factor enabling efficient OSC device function^3^. In such bound-polaron pair (BPP) states, the centre-of-charge distance ≥4 nm (~ the Coulomb capture radius)^25^ between the electron and the hole across the D/A interface can be expected to minimize the exponentially decaying exchange interactions, resulting in near degeneracy of the interfacial singlet and triplet states and thus potentially increasing the rate of spin-state mixing processes. Analogous nanosecond radical pair singlet-triplet mixing processes are well-documented for the cases of donor–acceptor molecules in solution^26^,^27^,^28^,^29^,^30^,^31^,^32^, polymer-polymer blends^8^,^9^, phthalocyanine-fullerene blends^16^,^17^ and photosynthetic reaction centres^33^,^34^. Indeed, the existence of such singlet–triplet mixing within bound electron-hole pair recombination pathways would have important implications for OSC optimization in terms of optimizing charge separation^3^, singlet oxygen induced photodegradation^5^ and singlet fission based strategies to enhance photocurrent densities^35^.

Provoked by this uncertainty over the exact dynamics of the electron-hole pair at the organic D/A interfaces and the importance of spin, we investigate two silaindacenodithiophene (SiIDT) copolymer/[6,6]-phenyl C71 butyric acid methyl ester (PC70BM) blend films, which show contrasting excited state energetics and solar cell device performance^36^. Using a combination of time-resolved laser spectroscopic techniques and quantum chemical calculations, we demonstrate that charge separation and polymer triplet generation compete on the nanosecond timescale only in the material system with a low driving energy for charge separation. The generation of polymer triplet excitons is shown to derive from back-recombination of triplet BPP states formed via spin-state mixing. In addition, electric field-dependent spectroscopy experiments reveal that the competition between charge separation and triplet generation dynamics is voltage dependent, thus suggesting possible routes for controlling the charge and spin dynamics at organic D/A interfaces.

## Results

### Optical and photovoltaic properties of blends

**Figure 1:**
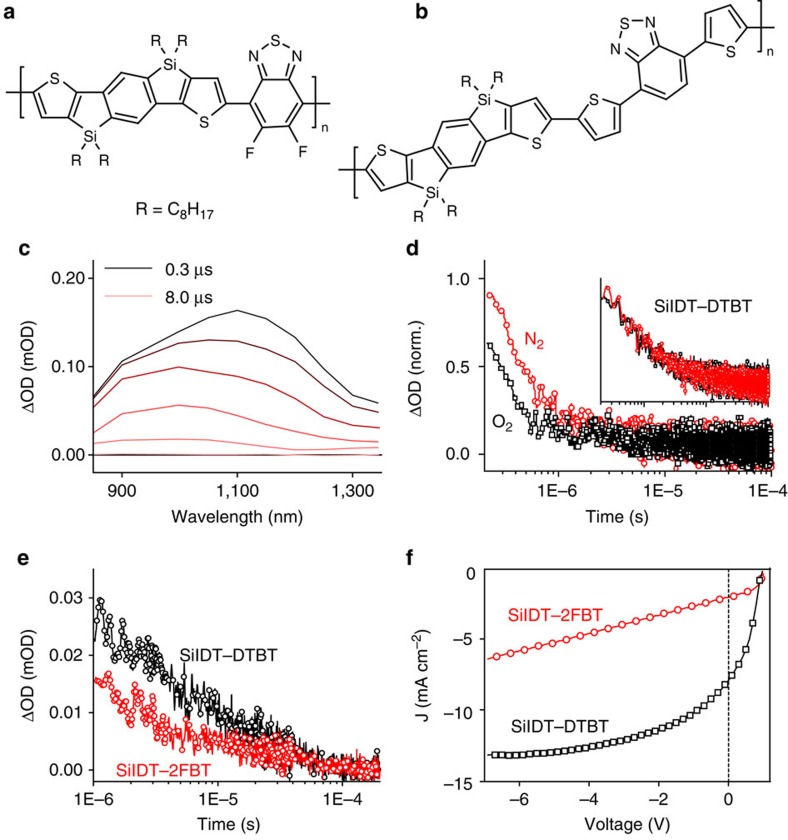
Microsecond TAS and device photocurrent densities. Chemical structures of the polymers studied herein: SiIDT–2FBT shown in (**a**) and SiIDT–DTBT shown in (**b**). Transient absorption spectra of the SiIDT–2FBT/PC70BM (1:2) blend film from 300 ns to 8 μs are shown in (**c**). The spectra were recorded using 630-nm 13-μJ cm^−2^ excitation pulses. The sample was kept under constant nitrogen flux. (**d**) Transient kinetics of the SiIDT–2FBT/PC70BM film recorded under oxygen and nitrogen atmospheres with a 1,150 nm probe and 630 nm 7.5 μJ cm^−2^ pump. The inset shows the kinetics recorded under oxygen and nitrogen for SiIDT–DTBT/PC70BM. (**e**) A comparison of the polaron transient absorption decays (normalized for photons absorbed) for SiIDT–2FBT/PC70BM (1:2) and SiIDT–DTBT/PC70BM (1:3) blends at 980 nm. This absorption is assigned to polarons because the triplet has decayed to near zero on the time scales plotted. (**f**) Reverse bias photocurrent density, corrected for dark injection, for the SiIDT–2FBT/PC70BM (1:2) and SiIDT–DTBT/PC70BM (1:3) devices. mOD, milli-optical density; nom., normal.

The polymers studied here are synthesized by co-polymerization of SiIDTwith 5,6-difluorobenzo[*c*][1,2,5]thiadiazole (2FBT) and 4,7-di(thiophen-2-yl)-benzo[*c*][1,2,5]thiadiazole (DTBT) following published procedures^36^. In SiIDT–2FBT, the BT unit is modified by the addition of two electron-withdrawing fluorine atoms, while in SiIDT–DTBT two thienyl spacers are added to give differing optical bandgaps (Supplementary Table 1) and HOMO levels of the two polymers^36^. In addition, an increased polymer film crystallinity was observed for SiIDT–2FBT compared with SiIDT–DTBT by X-ray diffraction (XRD) and transmission electron microscopy studies^36^. The energetic driving force for charge separation (Δ*E*_CS_), defined as the energy difference between the polymer singlet exciton and the nominal electrical gap of the blend (IP_Donor_−EA_Acceptor_), is estimated to be 0.3 eV smaller for SiIDT–2FBT/PC70BM (0.1 eV) than for SiIDT–DTBT/PC70BM (0.4 eV), based on PESA measurements^36^. This lower ΔE_CS_ for SiIDT–2FBT/PC70BM correlates with substantially (2–3 fold) lower short circuit photocurrent in solar cells with a device of structure indium-tin-oxide/PEDOT:PSS/polymer-fullerene/Ca/Al; this observation is analogous to energetic correlations with charge generation efficiency and photocurrent density that we and others have reported for other material combinations^4^,^37^,^38^. Figure 1f presents device current densities corrected for dark injection, while uncorrected device characteristics are included in Supplementary Fig. 12 and Supplementary Table 2. In addition, it is apparent from Fig. 1f that the two polymers have very different voltage dependent current profiles, indicating differences in the voltage dependence of their charge separation or recombination properties, as we discuss below.

### Monitoring charge dynamics and triplet state population

To study these differences in device performance, we first determine the polymer fluorescence quenching yields in the SiIDT–DTBT/PC70BM (1:3 weight ratio) and SiIDT–2FBT/PC70BM (1:2 weight ratio) blends using photoluminescence (PL) spectroscopy. The results show that both the blends have very high PL quenching yields >96% (see Supplementary Fig. 1). This indicates that exciton separation at the D/A interface does not limit photocurrent generation in the studied systems. Therefore, material mixing in the film active layer must be very intimate, with pure polymer domains (if present at all) exhibiting diameters substantially less than the exciton diffusion lengths, typically on the order of <10 nm for such polymers. In addition, the energy alignment at the polymer/fullerene interface must also be suitable for efficient electron transfer (exciton dissociation) across the interface. Nevertheless, despite efficient polymer exciton quenching for both the systems, photocurrent generation is clearly lower for the SiIDT–2FBT, indicative of recombination losses following exciton quenching limiting photocurrent for this system.

To address this issue further, transient absorption spectroscopy (TAS) on the nanosecond and microsecond time-regime is undertaken to estimate the charge separation yields of the two studied blends^39^. Thin films of SiIDT–DTBT/PC70BM and SiIDT–2FBT/PC70BM are prepared following reported device film fabrication procedures^36^. The samples are excited at the lowest polymer absorption maximum (630 nm) and the changes in film absorption are recorded in the NIR spectral region. Full data sets including those of neat polymer films are shown in Supplementary Figs 2 and 3. Figure 1c presents the transient absorption spectra of SiIDT–2FBT/PC70BM, where two absorption bands are noted, one at 1,000 nm and another at 1,100 nm. The absorption band at 1,100 nm dominates the spectra in the first microsecond, while the blue-shifted 1,000-nm absorption band is dominant at later delay times. The decay of the transient absorption signal at 1,150 nm measured under oxygen and nitrogen-rich environment is shown in Fig. 1d. This decay is strongly biphasic, and is fit to the sum of single-exponential and power law functions. Only the fast, exponential, decay phase is quenched by molecular oxygen. Therefore, the 1,100-nm absorption band, which bears similarity with the spectrum of the triplet exciton measured for the neat polymer (also with a maximum at 1,100 nm, see Supplementary Fig. 2) is assigned to absorption by polymer triplet excitons. The long-lived signal at 1,000 nm obeys a power law decay and is thus assigned, by analogy with previous studies, to the absorption by polymer polarons^40^,^41^. Negative polarons residing on PC70BM are not clearly resolved as they have low extinction coefficient and absorb at 1,350 nm (ref. 42). In addition, the lack of isosbestic points between the polaron and triplet absorption bands suggests that triplets and polarons decay independently from each other. Our observation of strong PL quenching in this system suggests the polymer triplets observed in the blend are not derived from direct intersystem crossing from singlet excitons, (as the PL data indicates these singlet states are strongly quenched) but rather are populated via a charge recombination process. Supporting this conclusion, we note that the triplet yield in the blend film at 300 ns is~300% higher than in the neat SiIDT–2FBT film (see Supplementary Fig. 2), indicating that this triplet generation via charge recombination proceeds in the blend with a high quantum yield.

In comparison to SiIDT–2FBT/PC70BM, the transient absorption spectra of SiIDT–DTBT/PC70BM consist only of a broad band peaking at 1,000 nm assigned to polaron absorption (see Supplementary Fig. 3). In addition, comparison of the data at 980 nm for the two different blends (Fig. 1e) shows that SiIDT–2FBT/PC70BM has smaller polaron yields on the microsecond timescale compared with SiIDT–DTBT/PC70BM (these data are normalized to the same densities of absorbed photons), consistent with the lower photocurrent densities observed in SiIDT-2FBT/PC70BM devices.

**Figure 2:**
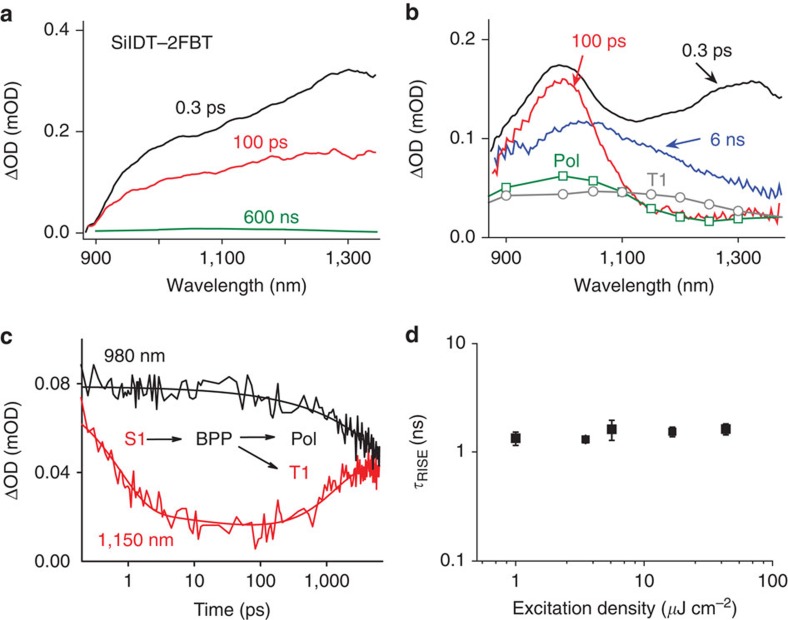
Charge and triplet state dynamics monitored with femtosecond transient absorption spectroscopy. (**a**) Transient absorption spectra of the SiIDT–2FBT film recorded at 0.3 ps, 100 ps and 600 ns, assigned to absorption by the polymer singlet exciton (S1). (**b**) Transient absorption spectra of the SiIDT–2FBT/PC70BM (1:2) film recorded at 0.3 ps, 100 ps and 6 ns. The 0.3-ps spectrum is a mix of polymer singlet exciton absorption (1,300 nm) and BPP state absorption (1,000 nm). The 100-ps spectrum is assigned to BPP absorption, while both polymer triplet and polaron absorption contribute to the spectrum at 6 ns. The polymer polaron (Pol) and triplet exciton (T1) absorption spectra are determined from microsecond data, as described in Supplementary Note 1. (**c**) Transient absorption signals as a function of time delay measured at 980 nm (primarily polaron absorption) and 1,150 nm (primarily singlet and triplet exciton absorption). (**d**) Time constant of triplet exciton generation as a function of excitation density, received from global fitting of the single wavelength kinetics at ~980, ~1,150 and ~1,300 nm for SiIDT–2FBT/PC70BM 1:2 weight ratio blends. Error bars represent s.d. received from the non-linear least square fitting. mOD, milli-optical density.

Having concluded that the SiIDT–2FBT/PC70BM blend, despite showing very efficient singlet exciton quenching, exhibits only a relatively low yield of long-lived polarons, but rather a significant yield of polymer triplet excitons, we employ ultrafast TAS to explore the origin of this behaviour. The excitation density used in the experiment is 1.0 μJ cm^−2^, corresponding to a density of photogenerated excitons ~10^17^ cm^−3^, close to the charge density extracted from the device 2.2 × 10^16^ cm^−3^ under normal operating 1 sun conditions^43^. In Fig. 2b, the TA spectra of SiIDT–2FBT/PC70BM at 300 fs, 100 ps and 6 ns are presented. The polymer polaron and triplet absorption spectra obtained from the microsecond TAS are also included in the graph. A neat PC70BM film was measured but did not show detectable signals at the relevant excitation densities. The TA spectra of the neat polymer are shown in Fig. 2a. The SiIDT–2FBT neat film has a broad transient spectrum with a maximum at 1,300 nm, which we assign to polymer singlet exciton absorption. The lifetime of the exciton is estimated to be ~160 ps by mono-exponential fitting of the signal decay (Supplementary Fig. 4). A very weak positive signal is noted at later times corresponding to the triplet exciton absorption. On the other hand, the SiIDT–2FBT/PC70BM blend film shows more complex spectral dynamics, initially dominated by the polymer singlet exciton absorption (peaking at 1,300 nm), which decays rapidly to leave a positive absorption band at 1,000 nm. This band at 1,000 nm has a similar absorption maximum and a spectral shape to that of the polymer polaron observed on microsecond time scales and is therefore assigned to SiIDT–2FBT polarons, although its full width half maximum is significantly smaller (as further discussed below). The spectral evolution from singlet exciton absorption to polaron absorption is therefore assigned to the charge generation from polymer excitons. A global kinetic analysis of this process yields a time constant of ~0.3 ps, apparent for example as the rapid decay phase in the single wavelength kinetics at 1,150 nm plotted in Fig. 2c. Such ultrafast exciton dissociation is consistent with our observation of very strong PL quenching. Following this ultrafast charge generation process, there is further spectral evolution over the following nanoseconds, with the 1,000 nm absorption band decaying in amplitude, while at longer wavelengths there is a pronounced increase in the transient absorption. The resultant red-shifted and broad absorption spectrum is similar to that observed at longer delay times, and thus assigned, as above, to a combination of polaron and triplet absorption. Global analysis of this process yields a time constant of ~1.0 ns (apparent in Fig. 2c as the decay of the polaron absorption at 980 nm) and growth in triplet absorption at 1,150 nm. This 1.0-ns process in the blend film is therefore assigned to polaron recombination to yield the SiIDT–2FBT triplet excitons.

Further global fitting analyses of the transient absorption data of SiIDT–2FBT/PC70BM (1:2) blends measured at different excitation densities shows that the rise-time of the polymer triplet and the decay of the polaron signals are essentially independent of excitation density, as shown in Fig. 2d (see Supplementary Fig. 5 for details). At high excitation densities >1*10^18^ cm^−3^ (>20 μJ cm^−2^), a modest acceleration of triplet generation is observed, indicative of triplet state population via fast nongeminate recombination (Supplementary Fig. 6), as previously reported by Rao *et al*.^3^ for PCDTBT-based devices. However, for lower excitation densities, more relevant to device operating conditions, the observed excitation density independence strongly suggests that the recombination process yielding these polymer triplets is a geminate rather than nongeminate process. Therefore, we conclude that the polymer triplets in this blend are populated via back-electron transfer from intermediate BPP states generated at the D/A interface by exciton separation. The assignment of the initially generated charges to BPP rather than dissociated charges can also be inferred by the relatively narrow full width half maximum of the 1,000 nm transient absorption band measured at 100 ps; the observed spectral peak broadening and red-shifting of the polaron peak at latter times would be expected for free polaron states^44^.

### Model for charge separation and triplet state generation

**Figure 3:**
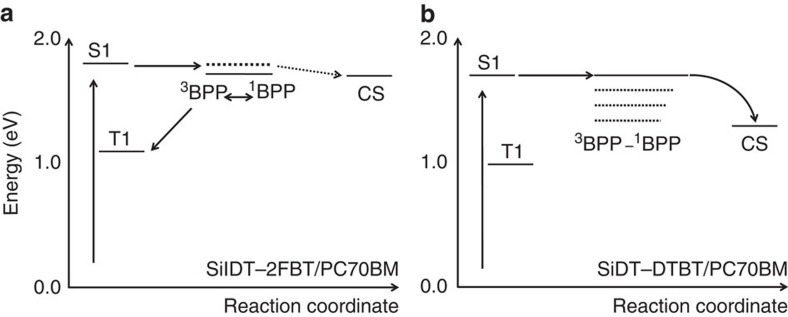
State diagrams depicting proposed model for charge separation and triplet generation. Models were developed based on our experimental observation of generation of polymer triplet excitons in SiIDT–2FBT/PC70BM as opposed to the observed generation of free charges in SiIDT–DTBT/PC70BM. (**a**) In SiIDT–2FBT/PC70BM, population of singlet ^1^BPP states is followed by intersystem crossing to degenerated triplet ^3^BPP states. Back-electron transfer from ^3^BPP then populates the lowest lying polymer triplet state (T1), thus limiting free charge generation in this system. (**b**) Model for charge separation in SiIDT–DTBT/PC70BM. S1 is the lowest energy polymer singlet exciton and CS is the charge separated state.

Figure 3a,b present a proposed model for charge separation and triplet generation for the SiIDT–DTBT/PC70BM and SiIDT–2FBT/PC70BM pairs, building upon previously published models for charge photogeneration and recombination in polymer/fullerene blends^4^,^18^. The energies for the singlet excitons of the polymers (S1) and the charge separated states (CS) are taken from experimental data as detailed above (and do not include entropic effects). The polymer triplet state energies were placed below the CS states in the two blends, based on the common observation that singlet–triplet energy splitting in polymers is ~0.6–0.7 eV (refs 45, 46). The energy difference between S1 and CS states is smaller for SiIDT–2FBT/PC70BM than SiIDT–DTBT/PC70BM. This model is consistent with previous experimental and theoretical work showing that the escape of charges from the polymer/fullerene junction is more efficient for materials with larger energy offsets^4^,^37^,^38^. For the SiIDT–2FBT/PC70BM blend, electron transfer across the interface creates Coulombically-bound polaron pair (BPP) states with a spin singlet character, in which the hole resides on the polymer chain, while the electron on the fullerene molecule. Motion of the polarons within their Coulomb attraction field is likely to reduce their charge-exchange integral thus creating energetically degenerate triplet and singlet bound states that can efficiently interconvert, enabled by hyperfine interactions^47^. Once generated, the triplet BPP states serve as precursors for subsequent efficient back-electron transfer to the lower lying polymer triplet excitons. In contrast, the SiIDT–DTBT/PC70BM blend, with a higher ΔE_CS_ is able to undergo efficient separation of charges, possibly via the formation of higher energy, more delocalized and therefore unbound charge-transfer (CT) states. This blend therefore avoids the formation of BPP states and the subsequent singlet-triplet intersystem crossing leading to geminate recombination to polymer triplets.

Time-dependent density functional theory calculations are undertaken to support and provide further insight into the model illustrated in Fig. 3. These calculations are performed on SiIDT–DTBT and SiIDT–2FBT oligomers and complexes of each oligomer with a PC70BM molecule for their singlet and triplet excitons and CT states. Details of the calculations are given in the Supplementary Note 2 and Supplementary Figs 7–10. The calculations confirm the energy alignments illustrated in Fig. 3, determining for example that for both oligomers, their triplet excitons are lower in energy than the singlet excitons and lower in energy than the oligomer/PC70BM CT states. The calculations indicate that the energy difference between the calculated singlet exciton and CT states results in higher energy, more delocalized charge-transfer states being energetically accessible from SiIDT–DTBT excitons, but not from SiIDT–2FBT excitons (Supplementary Table 3 and Supplementary Fig. 9). They also indicate that charge separation between oligomer and fullerene is more complete for SiIDT–DTBT/PC70BM than for SiIDT–2FBT/PC70BM^48^. These orbital considerations may be key reasons for the more complete charge separation in the SiIDT–DTBT/PC70BM blend^48^.

### Electric field-dependent generation of free charges

**Figure 4:**
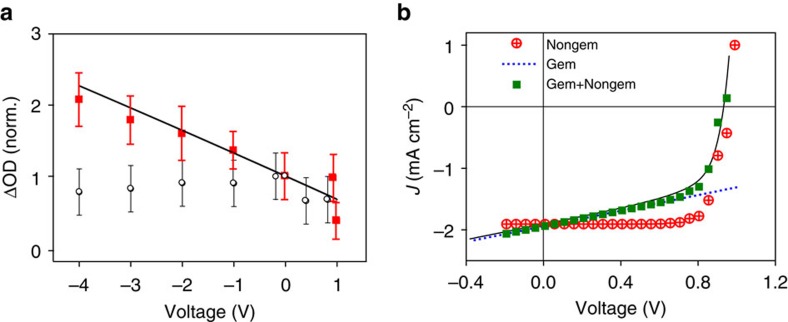
Charge yields as a function of bias and device JV reconstruction. (**a**) Field-dependent polaron yields plotted as a function of applied electrical bias of the SiIDT–2FBT/PC70BM 1:2 weight ratio device (red squares) and SiIDT–DTBT/PC70BM 1:3 wt. ratio device (black circles). The yields were estimated by averaging the transient polaron absorption signal decay from 0.5 to1.5 μs. Error bars represent the s.d. of fits of the transient absorption decays combined with the scaling uncertainty. The black line is the estimated field-dependent photocurrent generation profile of the SiIDT–2FBT/PC70BM device from (**b**). **b** Experimentally determined SiIDT–2FBT/PC70BM device *J–V* curve (black line) and the reconstructed *J–V* curve assuming nongeminate recombination photocurrent losses only (red circles), geminate and nongeminate losses (green squares). The blue broken line presents the field-dependent photocurrent loss in the device.

We now turn to test whether device macroscopic electric fields may be able to drive the separation of the BPP states observed for SiIDT–2FBT/PC70BM. Nanosecond TAS in a reflection mode of both SiIDT–DTBT/PC70BM (1:3 weight ratio) and SiIDT–2FBT/PC70BM (1:2 weight ratio) devices is carried out as a function of applied electrical bias. As shown in Fig. 4a, charge photogeneration is observed to be relatively independent of applied field for the SiIDT–DTBT/PC70BM device (transient absorption decays are provided in the Supplementary Fig. 11). This is consistent with relatively efficient charge generation in this blend even in the absence of macroscopic fields. In contrast, the SiIDT–2FBT/PC70BM device show a ~threefold increase in polaron generation yield under reverse bias. This observation suggests that the intermediate BPP states generated in this system can be dissociated under sufficiently large electric fields, thereby reducing the geminate recombination losses to the polymer triplets. To confirm this conclusion, we analyse further the device current density–voltage (*J*–*V*) behaviour of the SiIDT–2FBT/PC70BM device. First of all, we attempt to reconstruct the SiIDT–2FBT/PC70BM *J*–*V* curve (assuming a voltage-independent photocurrent generation) by the quantification of only nongeminate charge recombination losses in the device, determined by transient photovoltage (TPV) and charge extraction (CE) analyses (see Supplementary Note 3 and Supplementary Fig. 13 to 16 for details)^49^. These analyses indicate that nongeminate recombination losses at short circuit in this device are negligible. This reconstruction is clearly unable to recreate the experimental device *J*–*V* curve, as shown in Fig. 4b. In contrast, an excellent reconstruction of the *J*–*V* curve is obtained if we include a field-dependent photocurrent generation profile consistent with the transient absorption data in Fig. 4a (solid line)^49^,^50^. This confirms our conclusion that recombination of BPP states in the SiIDT–2FBT/PC70BM devices can be suppressed by the applied electric fields.

## Discussion

In summary, in this work we use femtosecond to millisecond transient absorption spectroscopies and a density functional theory calculation to study the charge photogeneration dynamics in SiIDT-based polymer/fullerene blends with differing energy offsets and film morphologies. For the blend with the lower energy offset, we observe ultrafast formation of BPP states that geminately recombine to the polymer triplets on a ~1-ns timescale. We propose a model for triplet generation, which involves the fast interconversion between the singlet and triplet BPP states at the D/A interface, with triplet BPP recombining to yield triplet polymer excitons. Field-dependent spectroscopy measurements and transient optoelectronic analysis indicate that the BPP formed in this blend can be dissociated by strong macroscopic electric fields. In contrast, the blend with the higher energy offset shows no evidence for BPP formation, nor triplet generation, but rather efficient field-independent charge generation, consistent with its almost threefold higher short circuit current density.

Our conclusion that polaron pairs formed at a D/A interface may undergo singlet–triplet spin conversion on a ~1-ns timescale, is relevant to recent discussion of the potential for spin-induced reduced recombination losses in organic photovoltaic devices. It suggests that such polaron pairs are likely to interconvert between spin states within the lifetime of these states, and therefore exhibit a behaviour independent of the spin multiplicity of the initially formed polaron pair. We further suggest this work is also relevant to the role of singlet–triplet polaron pair (or ‘charge transfer’) state mixing and triplet exciton generation at organic interfaces for other organic optoelectronic applications, including studies of organic magnetoresistance for memory applications and the maximization of electroluminescence yields in organic LEDs.

## Methods

### Film and device preparation

Films for spectroscopic characterization were spin-coated from polymer/PC70BM solutions (o-dichlorobenzene, 25 mg ml^−1^) with a weight ratio of 1:2 for SiIDT–2FBT/PC70BM and 1:3 for SiIDT–DTBT/PC70BM under standard conditions. For devices, the same blend compositions and spin-coating conditions were used. Indium–tin-oxide substrates with a sheet resistance of 15 Ωsq^−1^ (PsioTec Ltd, UK) were first cleaned in detergent, acetone and isopropanol and then oxygen plasma treated for 7 min at 100 W. The substrate was coated with filtered PEDOT:PSS and heated to 150 °C in air for 20 min on a hot plate. The active layer was then spin-coated in air and placed into a nitrogen glove box (<10 p.p.m. O2, H2O), where Ca (25 nm)/Al (100 nm) electrodes were deposited by thermal evaporation under vacuum. Device area was 0.045 cm^2^.

*J*–*V* curves were measured with a Keithley 238 source measure unit and a filtered 150-W xenon arc lamp generating simulated AM1.5 conditions (LOT Oriel). A calibrated reference silicon photodiode was used as a reference for the *J–V* measurements.

Ultraviolet/visible spectra of the thin films were measured with a PerkinElmer Lambda 25 spectrometer in air. The PL spectra were measured with a Fluorolog-3 spectrofluorometer (Horiba Jobin Yvon).

### Transient absorption spectroscopy

Submicrosecond and microsecond transient absorption kinetics were recorded using laser excitation pulses (<10 ns) generated from a tuneable optical parametric oscillator (Opolette 355). The light output of a tungsten lamp was used as a probe and signals were recorded with Si and InGaAs photodiodes, housed in a preamplifier and an electronic filter (Costronics Electronics) connected to a dual channel oscilloscope and PC. Probe wavelengths were selected with a monochromator. The films were kept under nitrogen atmosphere to prevent film degradation.

Femtosecond TAS was carried out using a commercially available transient absorption spectrometer, HELIOS (Ultrafast systems). Samples were excited with a pulse train generated by an optical parametric amplifier, TOPAS (Light conversion). Both, the spectrometer and the parametric amplifier were seeded with an 800 nm, <100 femtosecond pulses at 1 KHz generated by a Solstice Ti:Sapphire regenerative amplifier (Newport Ltd). Global analyses of the data were carried out using origin and Matlab.

### Device characterization

Charge extraction was performed at open circuit under different illumination intensities. In addition, for *J*–*V* reconstruction, the device voltage was varied using Keithley 2,400 sourcemeter, before extracting charges at short circuit. Devices were illuminated by a ring of white LEDs, where the LEDs are switched off (100 ns) and the device discharged close to short circuit over a measurement resistance of 50 Ω. The resulting transients were acquired with a TDS 3032 Tektronix digital oscilloscope, converted to a current using Ohm’s law and integrated with respect to time to calculate n.

Transient photovoltage was recorded in an open circuit under different illumination intensities, provided by a ring of white LEDs. A Nd:YAG pulsed laser (Continuum Minilite II) was used to generate small perturbations in the device and the resulting voltage transients were recorded with a TDS 3032 Tektronix digital oscilloscope and fitted with a single exponential function to obtain a carrier lifetime. Transient photocurrent was performed in a short circuit under different illumination intensities. Further details of the device modelling and characterization are included in the Supplementary Note 3.

## Author contributions

S.D.D. and J.R.D. designed and organized the project, developed interpretation of the results and wrote the manuscript. S.D.D. carried out optical characterization,TAS experiments and data analysis. H.U. carried out field-dependent spectroscopy measurements. S.W. carried out TPV and CE experiments and device *J–V* modelling. D.N., J.M.F. and J.N. carried out quantum chemical calculations and wrote the computational section in the Supplementary Information. B.C.S. and I.M. carried out the synthesis and design of the polymers. A. G. and P.S.T. fabricated the solar cell devices. S.D.D., J.R.D., S.W, J.N., J.M.F. and D.N. developed the interpretation of the results and supported the writing of the manuscript.

## Additional information

**How to cite this article:** Dimitrov, S. D. *et al*. Polaron pair mediated triplet generation in polymer/fullerene blends. *Nat. Commun.* 6:6501 doi: 10.1038/ncomms7501 (2015).

## Supplementary Material

Supplementary Information Supplementary Figures 1-16, Supplementary Tables 1-3, Supplementary Notes 1-3 and Supplementary References
