# Supplementary material for: Polaron pair mediated triplet generation in polymer/fullerene blends
Source: Nat Commun. 2015 Mar 4;6:6501. doi: 10.1038/ncomms7501 (PMC4366531; doi:10.1038/ncomms7501)
Supplement: Supplementary Information — Supplementary Figures 1-16, Supplementary Tables 1-3, Supplementary Notes 1-3 and Supplementary References [file ncomms7501-s1.pdf]

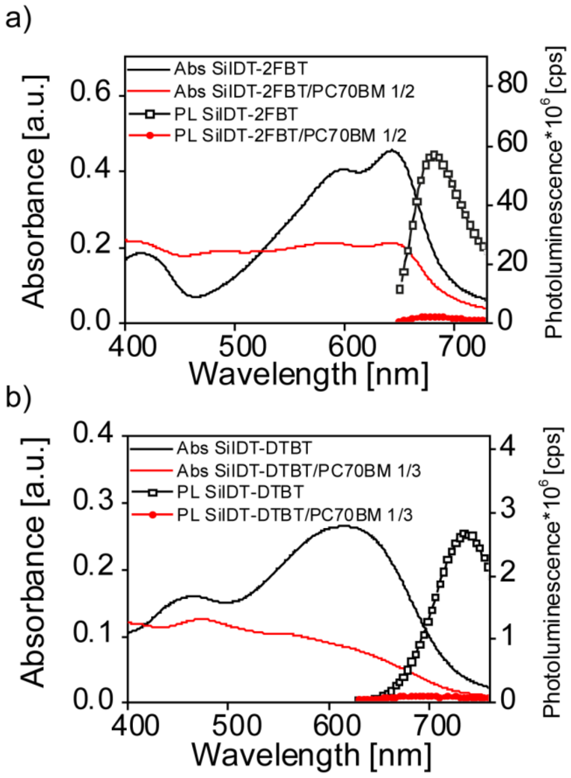

Supplementary Figure 1: Absorbance and photoluminescence spectra. UV/Vis absorbance and photoluminescence spectra of (a) SiIDT-2FBT and SiIDT-2FBT/PC70BM (1:2) thin films and (b) SiIDT-DTBT and SiIDT-DTBT/PC70BM (1:3) thin films. The films were prepared following published device film fabrication procedures.[1] Photoluminescence quenching by PC70BM in both blends was found to be ~96 %. The thickness of the films is 44±7 nm for SiIDT-DTBT/PC70BM and 67±5 nm for SiIDT-2FBT/PC70BM.

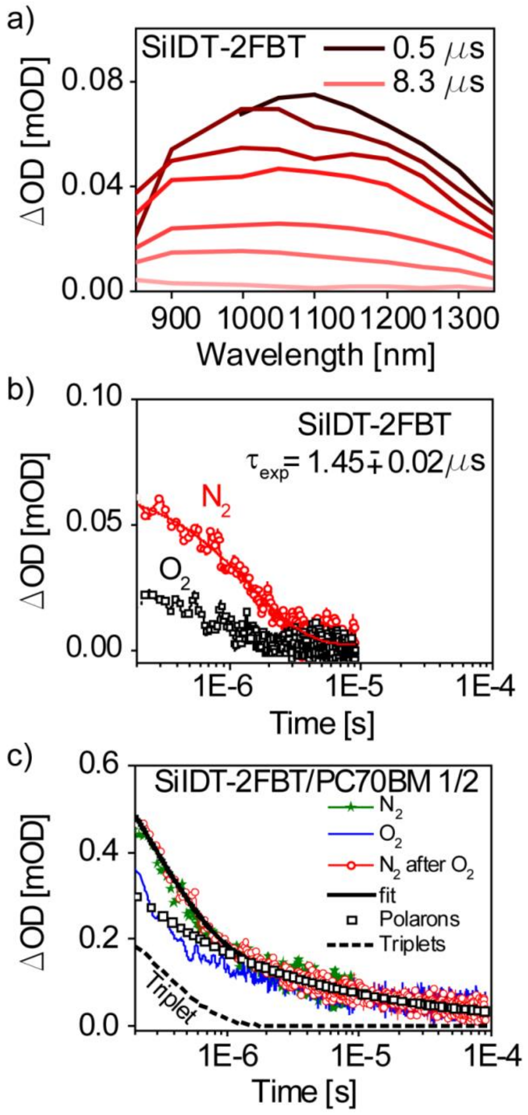

Supplementary Figure 2: Microsecond transient absorption spectroscopy of SiIDT2FBT and SiIDT2FBT/PC70BM. (a) Transient absorption spectra of thin SiIDT-2FBT film. Sample was excited with 635 nm laser pulses with 13.4 μJ.cm^{-2}. (b) Single wavelength kinetics of SiIDT-2FBT measured at 1000 nm with 635 nm, 3.1 μJ.cm^{-2} excitation under constant oxygen and nitrogen flux. (c) Single wavelength kinetics of SiIDT-2FBT/PC70BM (1:2) measured at 1000 nm with 635 nm, 3.1 μJ.cm^{-2} excitation. The kinetics were recorded under nitrogen (before and after oxygen measurements) and oxygen atmospheres. The signal amplitude and decay was found to be completely reversible when measured under a nitrogen atmosphere (green and red decays) after the oxygen quenching experiment (blue decay), thus indicating low sample degradation during the duration of our measurements. The kinetic (measured under N_2) was fitted with a $A + Bexp\left(\frac{-t}{\tau}\right) + Ct^n$ function to account for the mono-exponentially decaying polymer triplet exciton and the power law obeying polaron signal decay. The triplet contribution to the overall transient absorption signal was

thus extracted. A comparison of the triplet absorption at 300 ns after light excitation in the neat polymer and blend film shows that triplet generation in the blend is 3 times more efficient than in the neat polymer film.

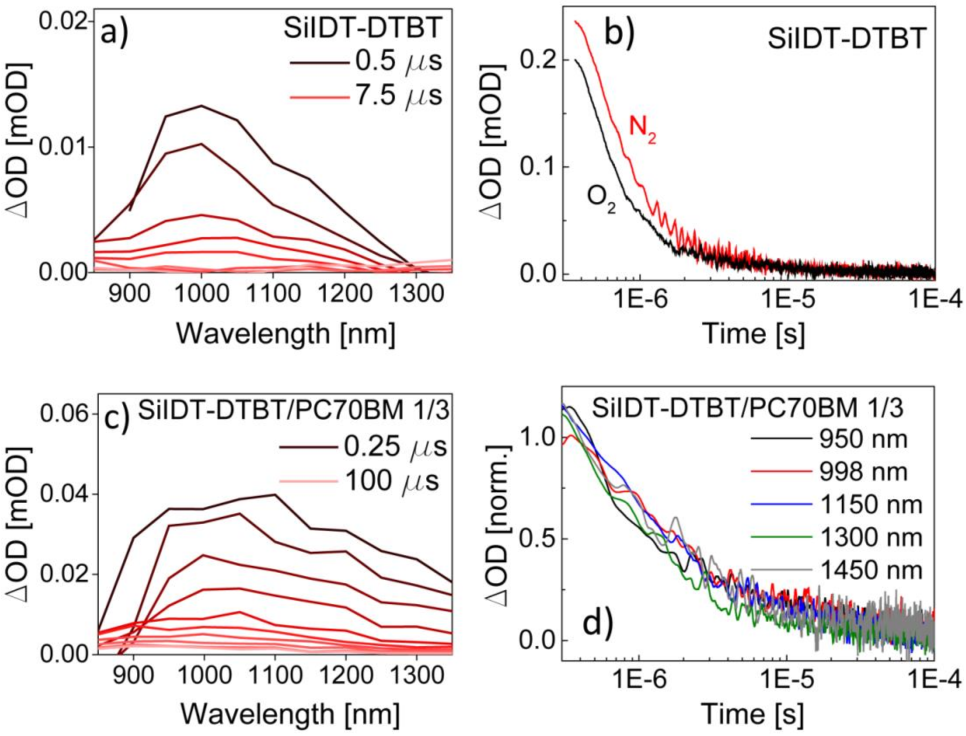

Supplementary Figure 3: Microsecond transient absorption spectroscopy of SiIDTDTBT and SiIDTDTBT/PC70BM. Transient absorption spectra of thin (a) SiIDT-DTBT and (c) SiIDT-2FBT/PC70BM (1:3) films. The films were prepared following device film fabrication procedures reported previously.[1] Laser excitation was with 630 nm laser pulses with an excitation density of 4.7 µJ.cm^{-2} for the neat film and 5.8 µJ.cm^{-2} for the blend film experiments. b) SiIDT-DTBT kinetics at 980 nm (630 nm excitation) in oxygen and nitrogen atmospheres show quenching of the signal in the presence of molecular oxygen. d) The SiIDT-DTBT/PC70BM (1:3) kinetics show that the signal decay is independent of the probed wavelength, thus indicating the presence of only polaron species in the film.

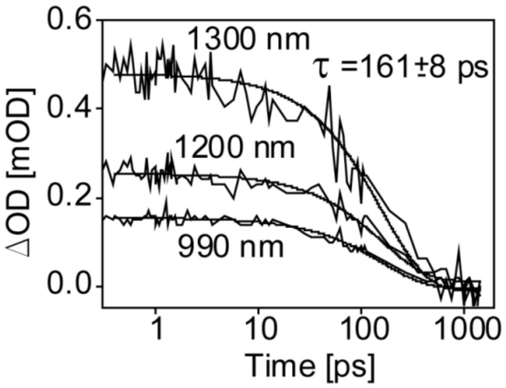

Supplementary Figure 4: Polymer singlet exciton decay. Transient absorption decays of the neat SiIDT-2FBT film excited at 635 nm and probed at 990, 1200, 1300 nm. The kinetics were fitted globally with a single exponential function yielding a 160 ps exciton lifetime.

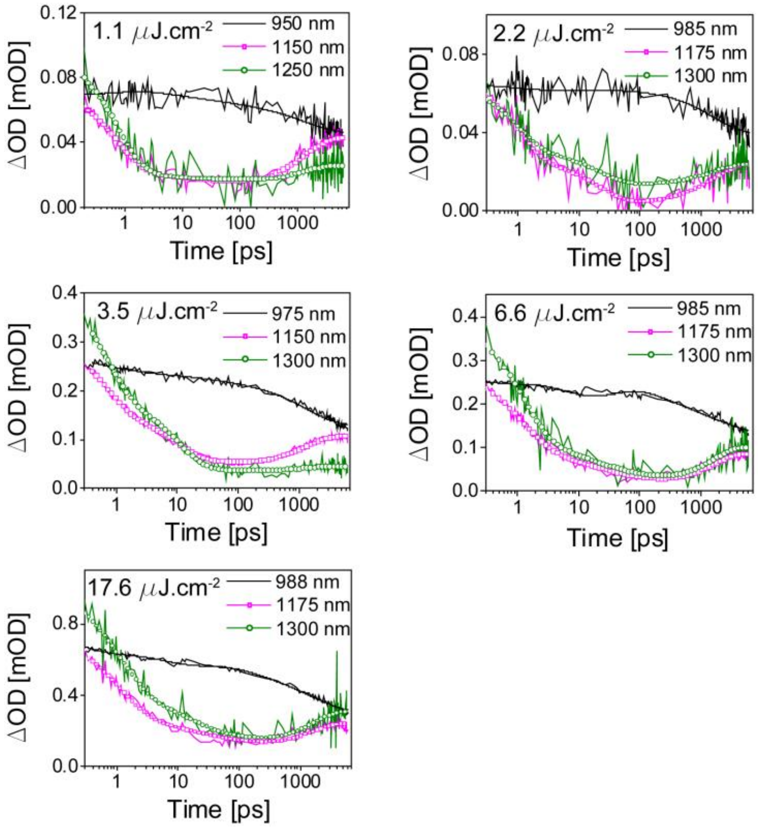

Supplementary Figure 5: Global fitting analyses of triplet and polaron decays. Global fitting analyses of the transient absorption data of SiIDT-2FBT/PC70BM (1:2) blends measured at different excitation densities. Kinetics were fitted with: $Aexp\left(\frac{-t}{\tau 1}\right) + Bexp\left(\frac{-t}{\tau 2}\right) + Cexp\left(\frac{-t}{\tau 3}\right) + Dt^{n}$. Data collected with higher excitation densities could not be fitted with this function and is not included here. The first two exponential terms correspond to the polymer singlet exciton decay, while the third exponent fits the rise time of the triplet signal starting at 100 ps.

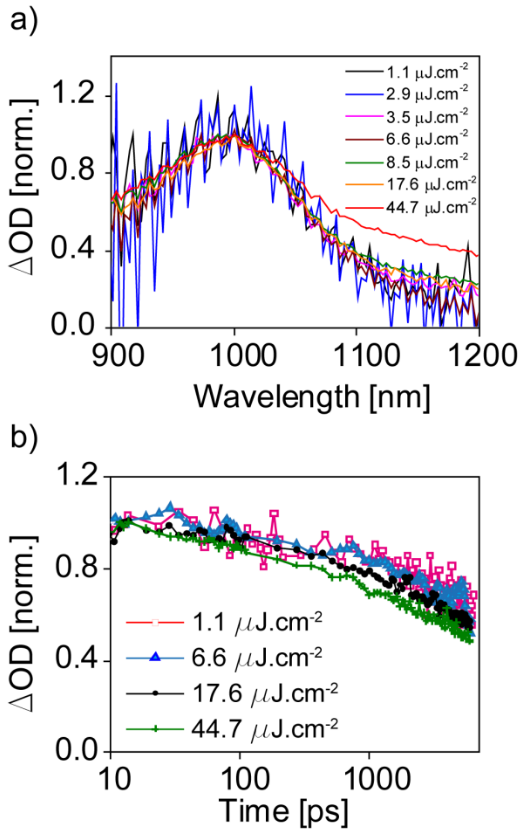

Supplementary Figure 6: Femtosecond transient absorption spectroscopy as a function of excitation density. a) Transient absorption spectra of the bound polaron pair in SiIDT-2FBT/PC70BM 1:2 wt. ratio film, recorded at 60 ps after 630 nm light excitation as a function of light intensity. b) decay of the transient absorbance of the bound polaron pair recorded at 990 nm as a function of light excitation density. The results show that polaron recombination is light intensity dependent only after excitation, with extremely high laser pulses generating on average more than 10^{18} excitons per cm^{-3}, indicative of weak charge density dependence of the polaron to triplet generation time constant.

a)

Conformer	Structure	Energy/trimer [eV]
SiIDT-2FBT ('wavy')	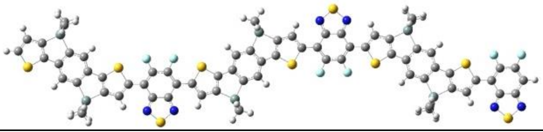	minimum
SiIDT-2FBT ('linear')	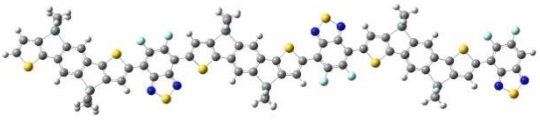	minimum+0.039
SiIDT-DTBT ('wavy')	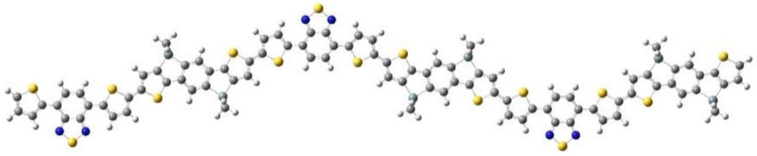	minimum
SiIDT-DTBT ('linear')	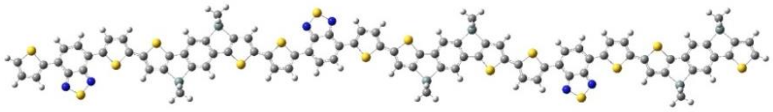	minimum+0.087

b)

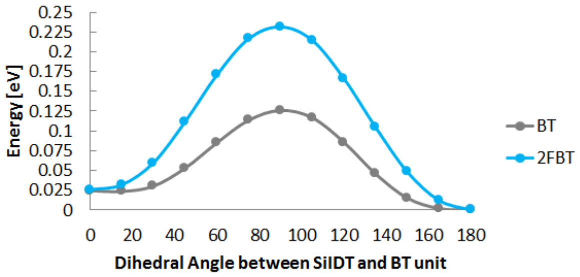

Supplementary Figure 7: Conformers and torsional potential energy surfaces of oligomers used in DFT studies. (a) Conformers of SiIDT-2FBT and SiIDT-DTBT copolymers and their energies calculated in vacuum at the DFT B3LYP/6-31G(d) level; 'minimum' indicates the most stable structure obtained from the full set of tested oligomer conformers. (b) Torsional potential calculated between SiIDT and BT (blue curve, higher amplitude) and between SiIDT and 2FBT (grey curve, lower amplitude).

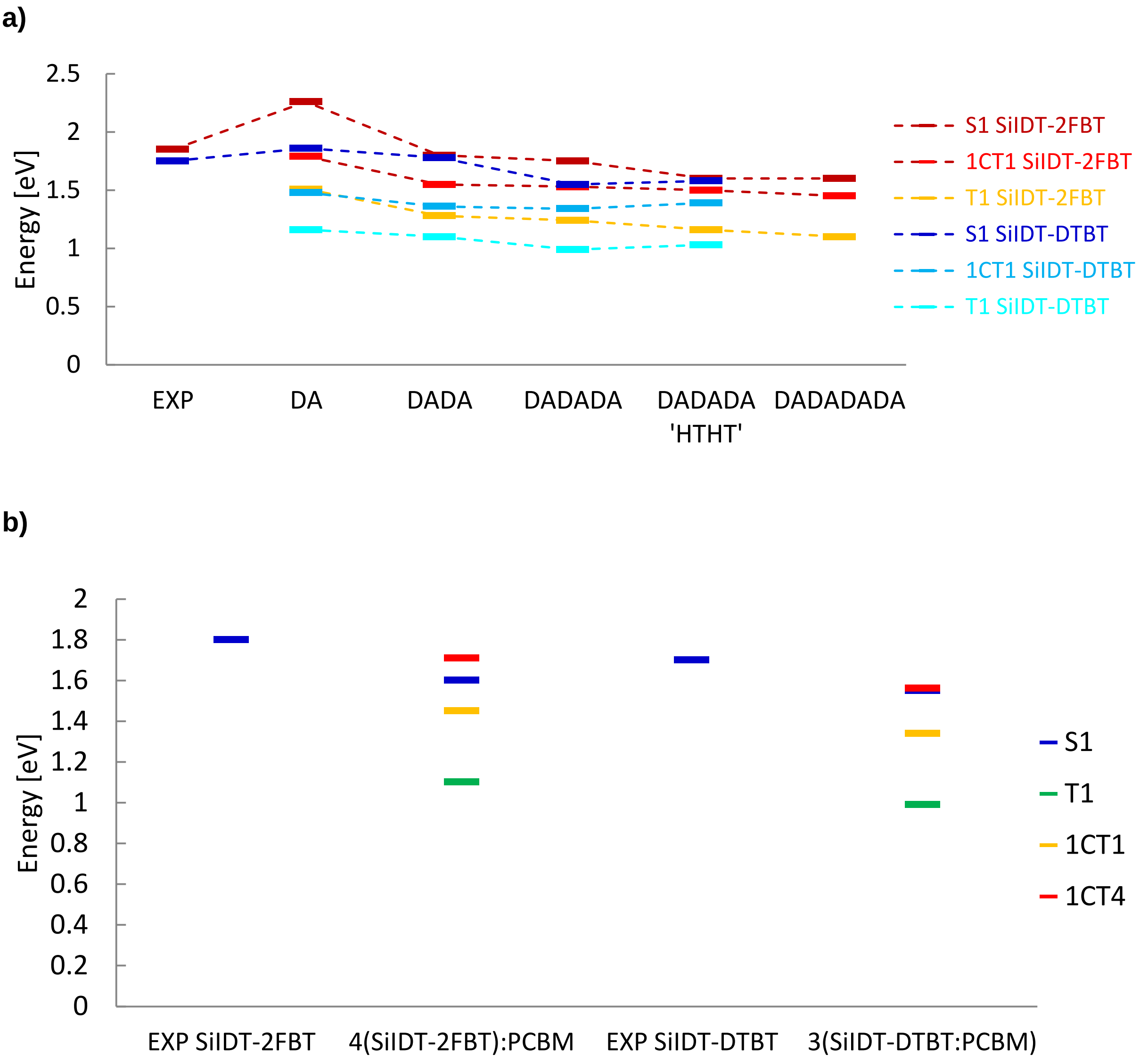

Supplementary Figure 8: Calculated energy level alignment of oligomers. (a) Size dependence of the calculated energy level alignment in the investigated blends obtained with TDDFT B3LYP/6-31G(d); (b) Comparison of the two model systems: tetramer of SiIDT-2FBT and trimer of SiIDT-DTBT blended with PC70BM; the experimental (EXP) values have been obtained from the onset of absorption of the neat polymer films, published in ref. 1. The S_1 (S1) and T_1 (T1) are the lowest–energy singlet and triplet excited states, respectively, calculated for an isolated oligomer. The $^1CT_1$ (1CT1) energy is the energy of the Coulombically-bound electron-hole pair across the interface. The triplet $^3CT_1$ state is not shown but its energy is calculated to be almost degenerate with the singlet $^1CT_1$.

a)

<table>
<tr><th>DADADA SiIDT-2FBT</th><th>Hole</th><th>Electron</th></tr>
<tr><td>S_1 (NTOs)</td><td>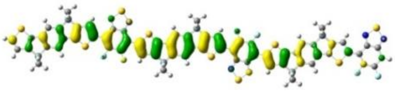</td><td>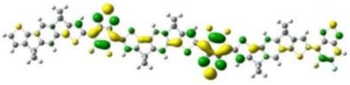</td></tr>
<tr><td>T_1 (spin density)</td><td colspan="2">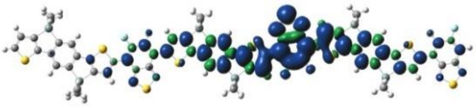</td></tr>
<tr><td>$^1CT_1$ (NTOs 0.005)</td><td>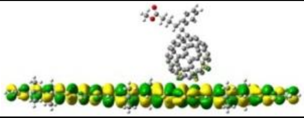</td><td>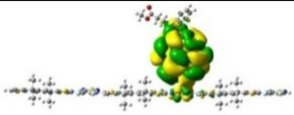</td></tr>
<tr><td>$^1CT_1$ (NTOs)</td><td>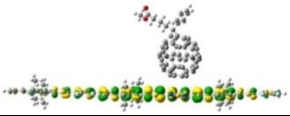</td><td>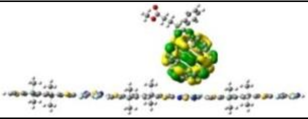</td></tr>
<tr><td>Polaron+ (MO)</td><td>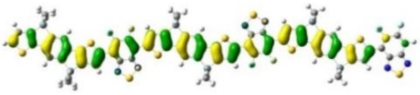</td><td></td></tr>
</table>

b)

<table>
<tr><th>DADADA SiIDT-DTBT</th><th>Hole</th><th>Electron</th></tr>
<tr><td>S_1 (NTOs)</td><td>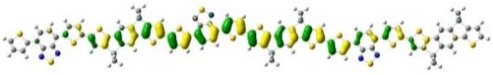</td><td>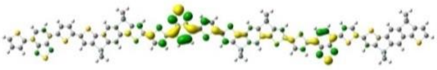</td></tr>
<tr><td>T_1 (spin density)</td><td colspan="2">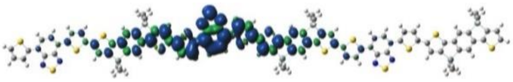</td></tr>
<tr><td>$^1CT_1$ (NTOs 0.005)</td><td>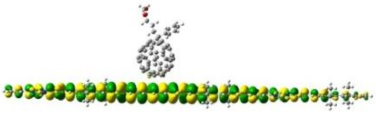</td><td rowspan="2">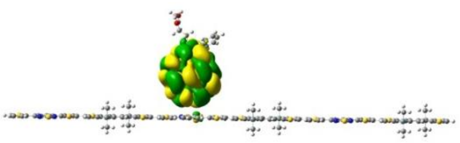</td></tr>
<tr><td>$^1CT_4$ (NTOs 0.005)</td><td>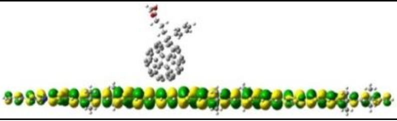</td></tr>
<tr><td>$^1CT_1$ (NTOs 0.02)</td><td>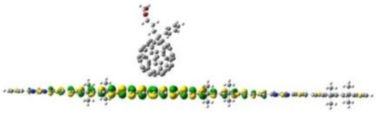</td><td rowspan="2">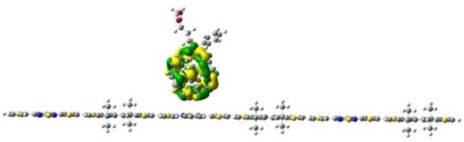</td></tr>
<tr><td>$^1CT_4$ (NTOs 0.02)</td><td>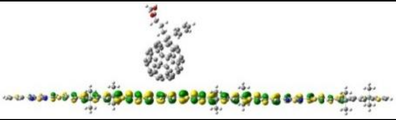</td></tr>
<tr><td>Polaron+ (MO)</td><td>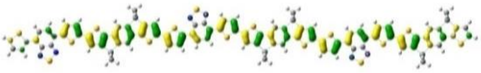</td><td></td></tr>
</table>

Supplementary Figure 9: Charger density isosurfaces. The density iso-values (calculated using a spin cut-off of 0.02 electronic-charge per Bohr^3 unless otherwise stated) of the lowest lying electronically excited states with charge transfer character ($^1CT_1$) in trimer:fullerene pairs as well as the hole polaron. (a) For SiIDT-2FBT the electron in the first excited state is delocalised between the oligomer and PC70BM. (b) For SiIDT-DTBT the electron and hole NTOs are localised on the PC70BM and oligomer units, respectively.

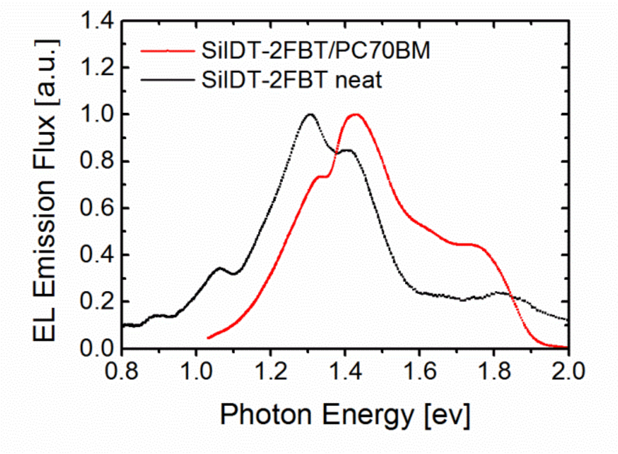

Supplementary Figure 10: Electroluminescence spectra Electroluminescence spectra of the films of SiIDT-2FBT and SiIDT-2FBT/PC70BM. The shift in energy between these two spectra was estimated to be 0.14 eV consistent with our quantum chemical calculations. Electroluminescence was measured using a spectrograph (Shamrock 303) combined with a InGaAs photodiode array (iDUS) cooled to -90 °C. Electroluminescence spectra from blend and pure polymer devices were measured at 11 mA/cm^2.

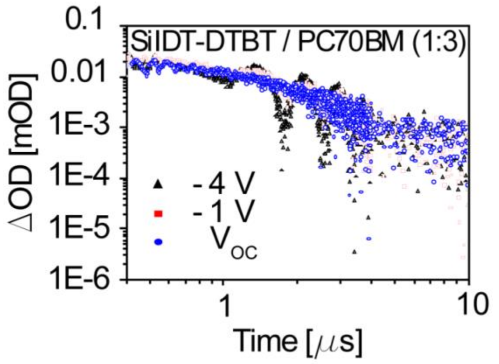

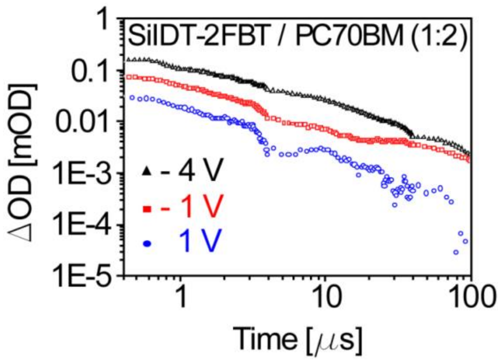

Supplementary Figure 11: Field dependent transient absorption spectroscopy. TA decays of SiIDT-DTBT/PC70BM (1:3) and SiIDT-2FBT/PC70BM (1:2) devices as a function of applied external electrical bias, measured with 635 nm and 630 nm excitation pulses, respectively. Probe pulses, 980 nm, were used to probe polaron absorbance.

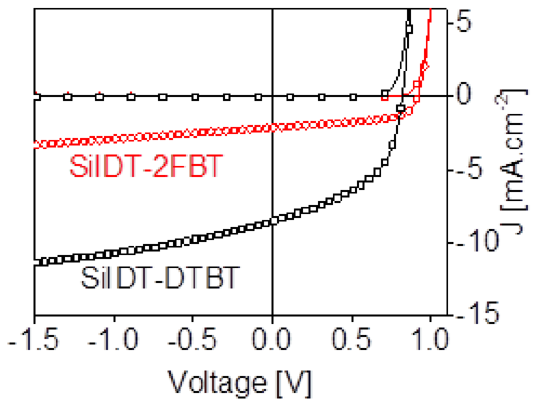

Supplementary Figure 12: Device JV curves. J-V curves of optimised SiIDT-2FBT/PC70BM (1:2) and SiIDT-DTBT/PC70BM (1:3) devices measured in dark and under AM1.5 one sun conditions.

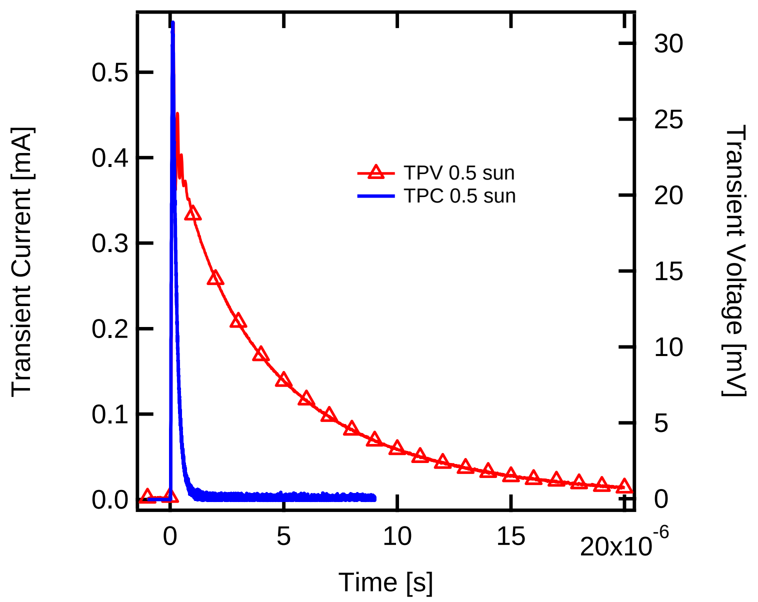

Supplementary Figure 13: Transient photocurrent and photovoltage decays. Transient photocurrent (line) and photovoltage (triangles) decays for SiIDT-2FBT/PC70BM (1:2) at 0.5 sun equivalent light intensity. Results show substantially faster decay time at short circuit suggesting efficient carrier extraction with little competition from non-geminate recombination.

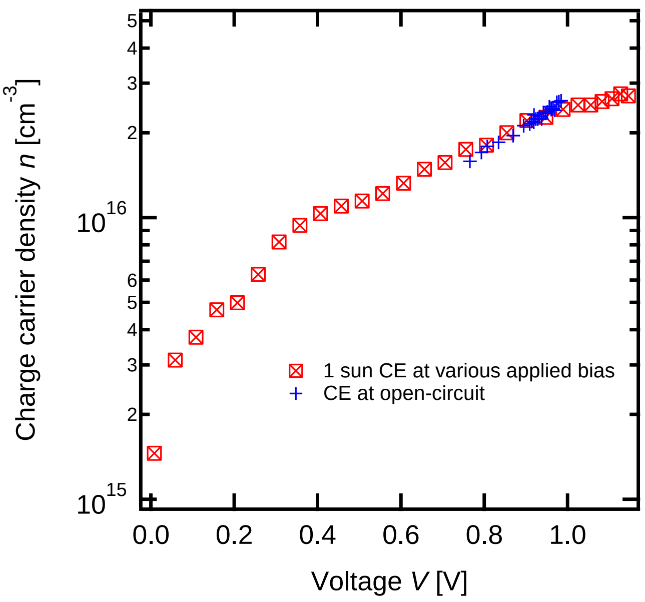

Supplementary Figure 14: Charge extraction. Charge extraction at open-circuit (crosses) and at 1 sun measured at various applied bias (squares) after correction for incurred charge carrier losses and capacitive charge on the electrodes for SiIDT-2FBT/PC70BM (1:2) device.

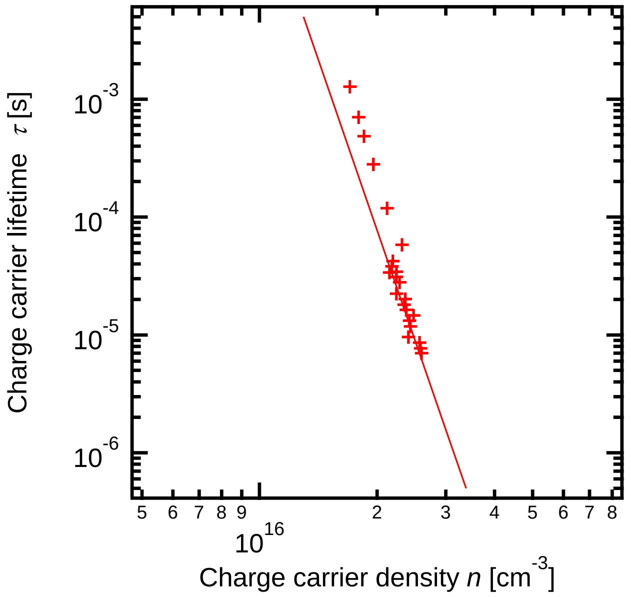

Supplementary Figure 15: Lifetime of charge carriers. Charge carrier lifetime as a function of charge carrier density measured at open-circuit for the SiIDT-2FBT/PC70BM (1:2) device.

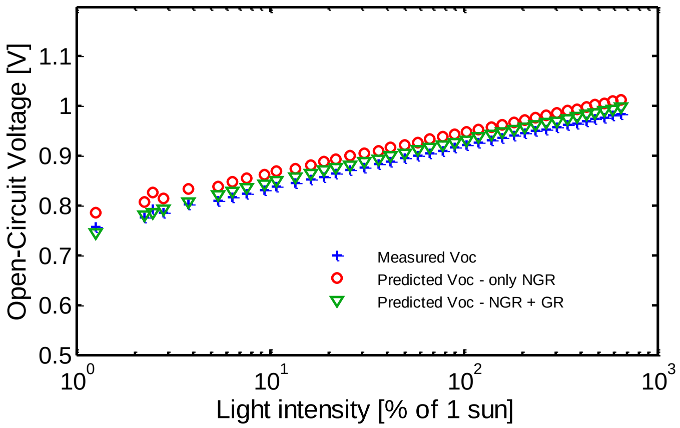

Supplementary Figure 16: Modelling of open circuit voltage. Predicted (blue crosses) and measured (red circles) open-circuit voltages as a function of light intensity for SiIDT-2FBT/PC70BM (1:2). The difference in predicted and measured V_{OC} at 1 sun is reduced to 11.0 mV when incorporating both field-dependent geminate recombination and non-geminate recombination compared to 27.3 mV with just non-geminate recombination. This is calculated from CE and TPV measurements of non-geminate recombination at open circuit such that $V_{OC} \approx \frac{1}{\gamma\delta} ln\left(\frac{(J_{SC}-J_{GR}(V_{OC}))\delta\tau_{\Delta n_0}}{edn_0}\right)$.

	S_1 EXP [eV][a]	E_{CS} PESA [eV][b]	S_1 CALC [eV]	E_{CT1} CALC [eV]	E_T CALC [eV]
SiIDT-2FBT/PC70BM	1.8	1.7	1.60	1.45	1.10
SiIDT-DTBT/PC70BM	1.7	1.3	1.55	1.34	0.99

Supplementary Table 1. Optical properties and experimental and calculated energy levels of SiIDT based polymers. (a) The energy of the lowest exciton transition (S_1) was estimated from the onset of absorption of the neat polymer films, published in ref. 1. (b) The energies of the separated charges were estimated previously using photoelectron spectroscopy (PESA)[1] and using 3.7 eV for the fullerene electron affinity.

	V_{OC} [V]	J_{SC} [mA.cm^{-2}]	FF	PCE [%]	Film thickness [nm]
SiIDT-2FBT/PC70BM	0.92	2.12	0.56	1.09	93
SiIDT-DTBT/PC70BM	0.82	8.52	0.49	3.45	75

Supplementary Table 2. Device characteristics. The device characteristics were determined from the J-V curves of the SiIDT-2FBT/PC70BM (1:2) and SiIDT-DTBT/PC70BM (1:3) blends.

SiIDT-2FBT/PC70BM	Length [Å]	S_1 [eV]	$^1CT_1$ [eV]	$^1CT_4$ [eV]	T_1 [eV]	$\|^1CT_1-S_1\|$ [eV]	$\|T_1-^1CT_1\|$ [eV]
EXP		1.8					
DA	~15	2.26	1.79	2.09	1.51	0.47	0.28
DADA	~30	1.80	1.55	2.02	1.28	0.25	0.27
DADADA (PC70BM translated ±2Å along from BT)	~50	1.75	1.53 (1.52)	1.81 (1.83)	1.24	0.22	0.29
DADADA 'wavy'		1.69	1.49	1.79	1.16	0.20	0.33
**DADADADA**	**~65**	**1.6**	**1.45**	**1.71**	**1.1**	**0.15**	**0.35**
SiIDT-DTBT/PC70BM	Length [Å]	S_1 [eV]	$^1CT_1$ [eV]	$^1CT_4$ [eV]	T_1 [eV]	$\|^1CT_1-S_1\|$ [eV]	$\|T_1-^1CT_1\|$ [eV]
EXP		1.7					
DA	~20	1.86	1.48	2.05	1.13	0.38	0.35
DADA	~40	1.78	1.36	1.71	1.10	0.42	0.26
**DADADA** (PC70BM translated ±2Å along from BT)	**~70**	**1.55**	**1.34** (1.32)	**1.56** (1.56)	**0.99**	**0.21**	**0.35**
DADADA 'wavy'		1.58	1.39	1.6	1.03	0.19	0.36

Supplementary Table 3. Excited state energies and energy differences for different oligomer sizes. For each trimer two additional oligomer-fullerene configurations were considered: (i) PC70BM displaced 2 Å along the oligomer axis (value in brackets for the $^1CT_1$), and (ii) a 'linear' oligomer replaced with the wavy Head-to-Tail conformer ('wavy').

Supplementary Note 1. Polymer and triplet exciton spectra. The polymer triplet absorption spectrum, included in Figure 2b was obtained from the microsecond transient absorption spectra of the neat polymer film as shown in Supplementary Figure 2. The polymer polaron spectrum in Figure 2b was obtained from the blend transient absorption spectrum recorded after 3 microseconds time delay in which the polymer triplet absorption after 3 microseconds is assumed to be negligible.

Supplementary Note 2. Quantum chemical calculation of excited state energetics. We have used time-dependent density functional theory (TDDFT) with the B3LYP functional to calculate relevant excited states of SiIDT-2FBT, SiIDT-DTBT and the two polymers combined with PC70BM. Both the energetics of these states relative to the ground state, and further analysis on the charge distribution for these states provide information on the processes occurring following photoexcitation of the two different polymers. Our model system is an oligomer interacting with a single PC70BM molecule in vacuum.

We have performed calculations on a tetramer of SiIDT-2FBT and a trimer of Si-IDT-DTBT (~65 Angstrom versus ~70 Angstrom end to end). Oligomers of such length provide excited state energetics (linear response TDDFT with B3LYP/6-31G(d)) comparable to the experimental data (Supplementary Figure 8 and Supplementary Table 3) at a modest computational cost. Furthermore, for such oligomer lengths we observe saturation to within ~ 0.05 eV in the oligomer length dependent values of the first singlet, triplet and charge transfer state energies.

Fullerene-oligomer pairs were constructed in a three step process. Firstly, the ground state geometry of the oligomer with methyl groups replacing the lateral alkyl chains was optimised at the DFT (B3LYP/6-31G(d)) level.[2] The calculated torsional potential between the SiIDT and 2FBT units (Supplementary Figure 7b) indicates that the optimum ground-state conformation is planar, with the barrier having risen to 25 meV at ±20 degrees from planarity in the gas phase (suggesting thermal fluctuations in torsion of ±20 degrees at 300 K). For non-fluorinated SiIDT-BT the torsional potential is shallower, allowing thermal fluctuations of ±40 degrees. This difference can be attributed to additional non-bonding interactions present between the fluorine atoms on the BT unit and sulphur or C-H groups on the SiIDT moiety.[3] The torsional potential for the SiIDT-DTBT similarly has a minimum for planar structures and is shallower than that for Si-IDT-2FBT. Both polymers can potentially form a variety of different conformations that are compatible with the optimum planar structure. We consider just two, (1) where the two thiophene units or fragments flanking the BT are both oriented so that their sulphur atoms point away from the thiodiazole unit, which we denote as 'wavy' and

(2) where the two thiophene units or fragments flanking the BT are oriented in opposite directions, which we denote as ‘linear’. The particular structures studied are shown in Supplementary Figure 7. Although for both systems the ‘wavy’ conformer is found to be more stable from the gas phase calculations, we choose to consider henceforth only the ‘linear’ conformers. We select these structures because the linear conformers are better able to organise into ordered domains, as required from the observed tendency of SiIDT-2FBT to crystallise and because the difference in the gas phase energies relative to the minimum energy ‘wavy’ conformers is only 0.013 and 0.026 eV per repeat unit for SiIDT-2FBT and SiIDT-DTBT, respectively. The higher tendency of SiIDT-2FBT to crystallise is probably influenced by the planarisation induced by fluorination.

In the next stage, the structure of the PC70BM molecule is optimised, using DFT with B3LYP/6-31G(d), and then placed in the space between the side chains of the optimised oligomer and above the acceptor (BT) unit in such a way that a hexagonal facet of the PC70BM is almost cofacial (i.e. slip-stacked orientation) with the 6-atom (benzoid) ring of the BT. The edge to edge oligomer-fullerene separation is set to 3.5 Angstroms. This is informed by a separate geometry optimisation with the ωB97XD functional. This functional contains Grimme's empirical dispersion correction, and so should produce better inter-molecular separations. Finally TDDFT calculations (B3LYP/6-31G(d)) are carried out on each fullerene-oligomer complex of B3LYP-optimised oligomer and PC70BM, as well as the isolated oligomers. The choice of a moderately sized basis set with additional polarisation functions helps to avoid overestimation of the excited state energies through basis set of limited size, while making calculations of this size (in number of atoms) computationally tractable.

We now investigate the excited state energy level alignment of the different systems (Supplementary Figure 8 and Supplementary Table 3**)** in order to understand why long-lived charge pair generation appears to be less efficient for SiIDT-2FBT:PC70BM blends than for SiIDT-DTBT in spite of favourable microstructure properties (high polymer crystallinity and intercalation of PC70BM between the polymer side chains). We aim to address two important mechanisms with regard to charge separation and device performance: (i) the role of ‘excess energy’ ($|^1CT_1-S_1|$, Supplementary Figure 8b) inherited from exciton generation upon light absorption and (ii) the possibility that the charge-transfer excited state recombines into a neutral triplet excited state in one of the two materials ($|T_1-^1CT_1|$).

First, we notice that in both cases the driving force for charge separation via the lowest $^1CT_1$ states, quantified as the difference between the first singlet and the lowest CT state, 1S-^{1}CT1, is small (0.21 eV for SiIDT-DTBT and 0.15 eV for SiIDT-2FBT) (note that for P3HT it is

~0.9 eV)[4]; however this driving force is slightly higher (by ~0.06 eV) for the SiIDT-DTBT:PC70BM system. In addition, for both model systems the triplet energy (T_1) is significantly lower (by 0.35 eV in both systems) than the lowest CT state ($^1CT_1$). Both the small 1S-^{1}CT1, energy and the substantial ^{1}CT1-1T energy are detrimental as they tend to limit charge separation and favour recombination to triplets. However, there is one feature that differs in the two systems: for SiIDT-DTBT:PC70BM the first higher lying ('hot') CT-state (namely $^1CT_4$) is almost resonant (~0.01 eV higher) with the lowest oligomer singlet (S_1), while for SiIDT-2FBT:PC70BM the $^1CT_4$ state lies 0.1 eV higher and is therefore less accessible energetically from the S1. The energy alignment of the S_1 and $^1CT_1$ remains almost unaltered when sliding the PC70BM by 2 Å away from the initial position along the polymer chain (the $^1CT_1$ changes by ~0.01 – 0.02 eV) for both systems. Selecting the 'wavy' conformer of the oligomer changes the energies of the singlet and triplet states, but it does not change the relative energy alignments of the crucial excited states.

A first qualitative understanding of the nature of the excited states is obtained by visualising the hole and electron natural transition orbitals (NTOs) of the vertical excitations calculated with TD-DFT B3LYP/6-31G(d). These orbitals can provide information on the charge transfer character and the degree of delocalisation of the excitation. In the case of SiIDT-2FBT the electron NTO of the $^1CT_1$ state has a noticeable contribution from the oligomer acceptor (BT) unit (Supplementary Figure 9a). This is not the case for SiIDT-DTBT:PC70BM, where the additional thiophenes on both sides of the BT unit reduce its electron withdrawing character, allowing the electron to localise on the fullerene (Supplementary Figure 9b).

Importantly, the hole wavefunction of the $^1CT_4$ state is more delocalised than in the $^1CT_1$ state in each case, so that the average electron-hole separation is higher when the system lies in the 'hot' rather than the 'cold' CT states. The delocalisation of the hole density along the oligomer backbone can be expected to reduce the net Coulomb interaction and so improve charge separation. This advantage would only apply in the SiIDT-DTBT:PC70BM system since the hot state is not accessible from the singlet of the SiIDT-2FBT.

Supplementary Note 3. Device JV reconstruction. Charge extraction (CE) was performed at open-circuit under different illumination intensities and at various applied bias, as described previously,[5] to measure the average excess charge carrier density (*n*) within the device relative to 0 V in the dark. The device is illuminated by a ring of white LEDs, which can achieve illumination intensities up to ~7 suns, for approximately 100 ms to allow the device to reach steady state conditions. The LEDs are switched off (100 ns) and the device discharged close to short-circuit over a measurement resistance of 50 Ω. The resulting

transients are acquired with a TDS 3032 Tektronix digital oscilloscope, converted to a current using ohms law and integrated with respect to time to calculate *n*. This is corrected from the capacitive charge on the electrode and recombination losses during extraction. *n* is observed to increase exponentially with open-circuit such that $n = n_0 e^{\gamma V_{OC}}$, where n_0 and γ are experimentally derived constants which describe the voltage dependence of average charge carrier density.

Transient photovoltage (TPV) was performed at open-circuit under different illumination intensities, as described previously,[5] to measure the average charge carrier lifetime. The device is held under continuous illumination provided by a ring of white LEDs. A small perturbation from a Nd:YAG pulsed laser (pulse duration 1-5 ns) is used to generate a small amount of extra charge in the device, which is forced to recombine under open-circuit conditions. The resulting voltage transient is measured with a TDS 3032 Tektronix digital oscilloscope and fitted with a single exponential function to obtain a carrier lifetime. This small-perturbation carrier lifetime is observed to vary exponentially with open-circuit such that $\tau_{\Delta n} = \tau_{\Delta n_0} e^{-\beta V_{OC}}$ where $\tau_{\Delta n_0}$ and β are experimentally derived constants which describe the voltage dependence of the average charge carrier lifetime. The small perturbation carrier lifetime can be related to the total charge carrier lifetime $\tau_n = \delta \tau_{\Delta n}$ where $\delta = \left(\frac{\beta}{\gamma} + 1\right)$ is the order of recombination.[6]

Transient photocurrent was performed at short-circuit under different illumination intensities, as described previously. Through the method of differential charging it is also possible to obtain the charge carrier density at open-circuit.[5]

The J-V curve can be described as the competition between a generation flux (J_{gen}) and recombination current (J_{loss}) such that $J(V) = J_{gen} - J_{loss}(V)$. Assuming field independent generation and no non-geminate recombination at short circuit, we make the approximation $J_{gen} \approx J_{SC}$. Assuming only non-geminate losses as measured by CE and TPV we calculate $J_{loss}(V)$ using:

Supplementary equation 1: $J_{loss} = J_{NGR} = ed\frac{n}{\tau_n} = \frac{edn^{\delta}}{\delta \tau_{\Delta n_0} {n_0}^{(\delta-1)}}$

where *e* is the electronic charge, *d* is the active layer thickness and δ, $\tau_{\Delta n_0}$ and n_0 are experimentally derived constants defined previously. As shown in Figure 4b the resulting J-V reconstruction is a poor match to the experimental data. For the field dependence observed in SiIDT-2FBT/PC70BM, a field dependent geminate recombination J_{GR} is included in the generation term, such that $J_{gen}(V) \approx J_{SC} - J_{GR}(V)$, where J_{GR} has the form of a quadratic. In

order to convert directly between optical density ΔOD measured in field dependent TAS and current density, the generation profile was referenced relative to J_{SC} at 0.5 sun illumination and scaled linearly with light intensity as previously described by Credgington *et al.*[6]

Supplementary References:

1 Schroeder, B. C. *et al.* Silaindacenodithiophene-based low band gap polymers - the effect of fluorine substitution on device performances and film morphologies. *Adv. Funct. Mater.* **22,** 1663-1670 (2012).

2 Few, S., Frost, J. M., Kirkpatrick, J. & Nelson, J. Influence of chemical structure on the charge transfer state spectrum of a polymer:fullerene complex. *J. Phys. Chem. C* **118,** 8253-8261 (2014).

3 Bronstein, H. *et al.* Effect of Fluorination on the properties of a donor-acceptor copolymer for use in photovoltaic cells and transistors. *Chem. Mat.* **25,** 277-285 (2013).

4 Veldman, D., Meskers, S. C. J. & Janssen, R. A. J. The energy of charge-transfer states in electron donor-acceptor blends: insight into the energy losses in organic solar cells. *Adv. Funct. Mater.* **19,** 1939-1948 (2009).

5 Shuttle, C. G. *et al.* Experimental determination of the rate law for charge carrier decay in a polythiophene: fullerene solar cell. *Appl. Phys. Lett.* **92,** 093311 (2008).

6 Credgington, D., Jamieson, F. C., Walker, B., Thuc-Quyen, N. & Durrant, J. R. Quantification of geminate and non-geminate recombination losses within a solution-processed small-molecule bulk heterojunction solar cell. *Adv. Mater.* **24,** 2135-2141 (2012).
